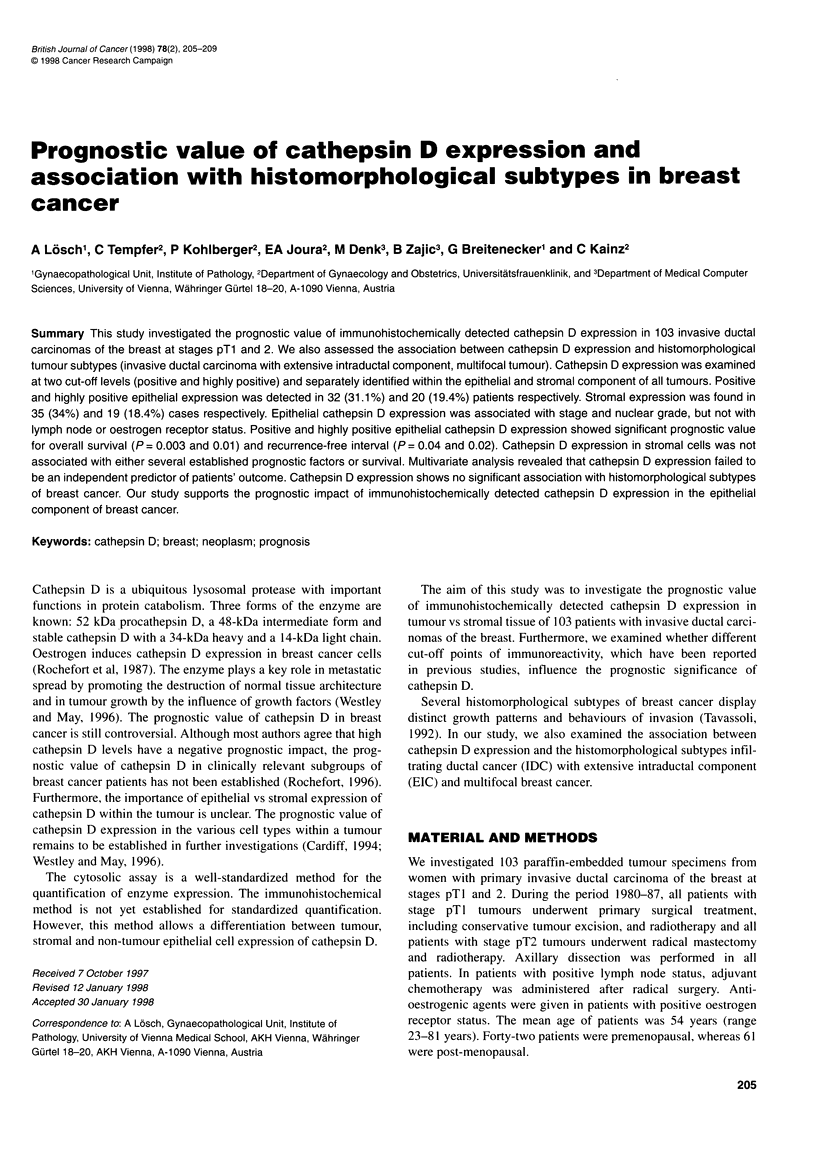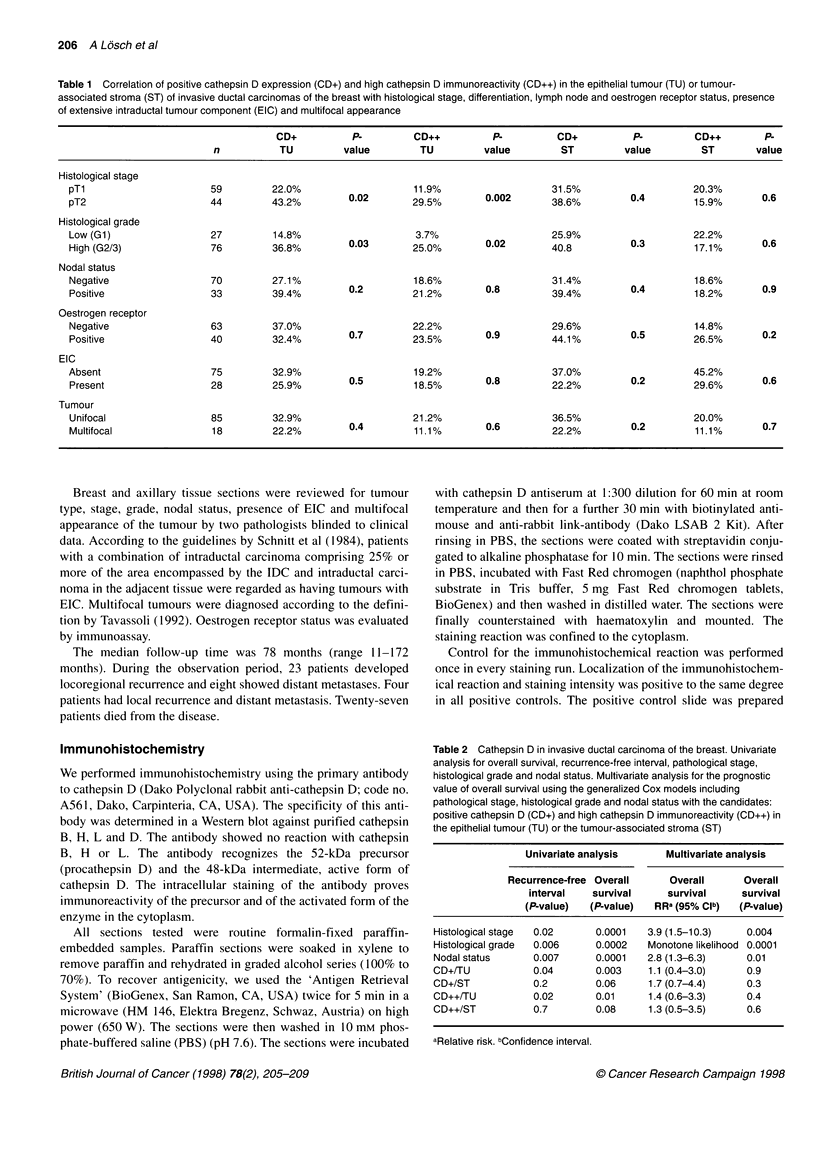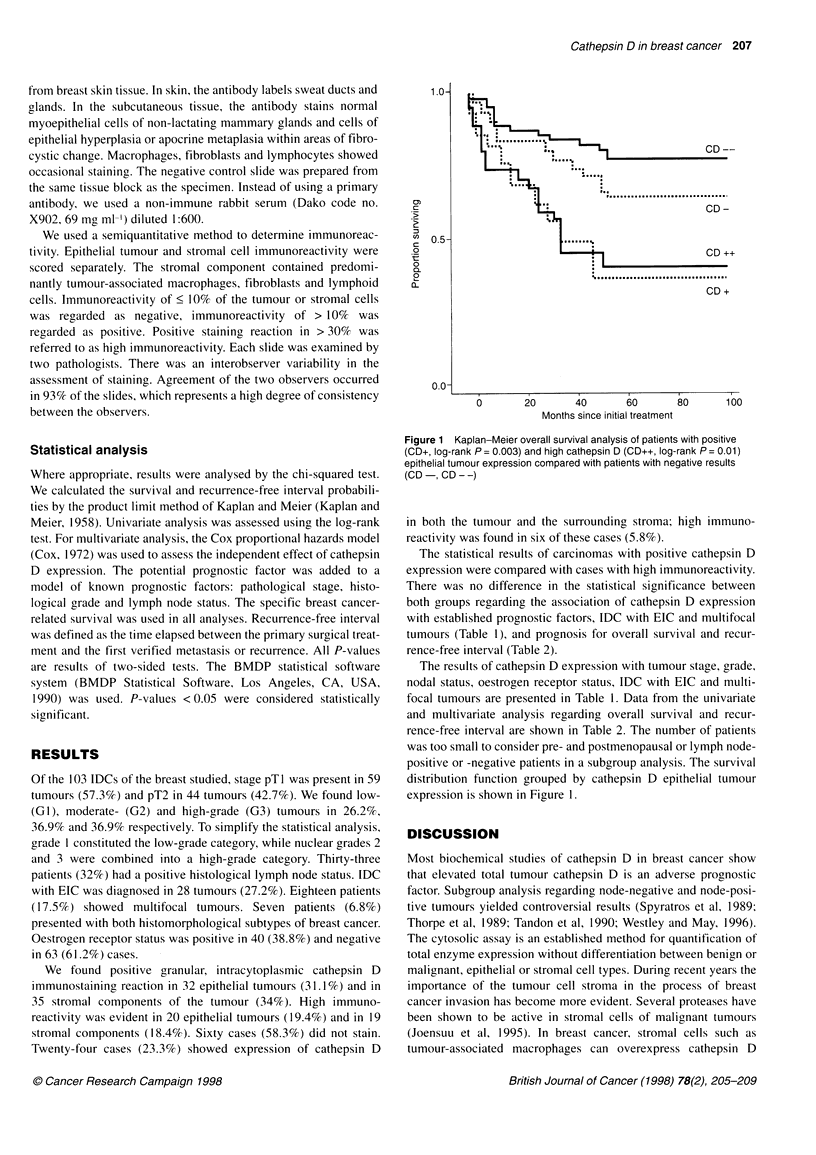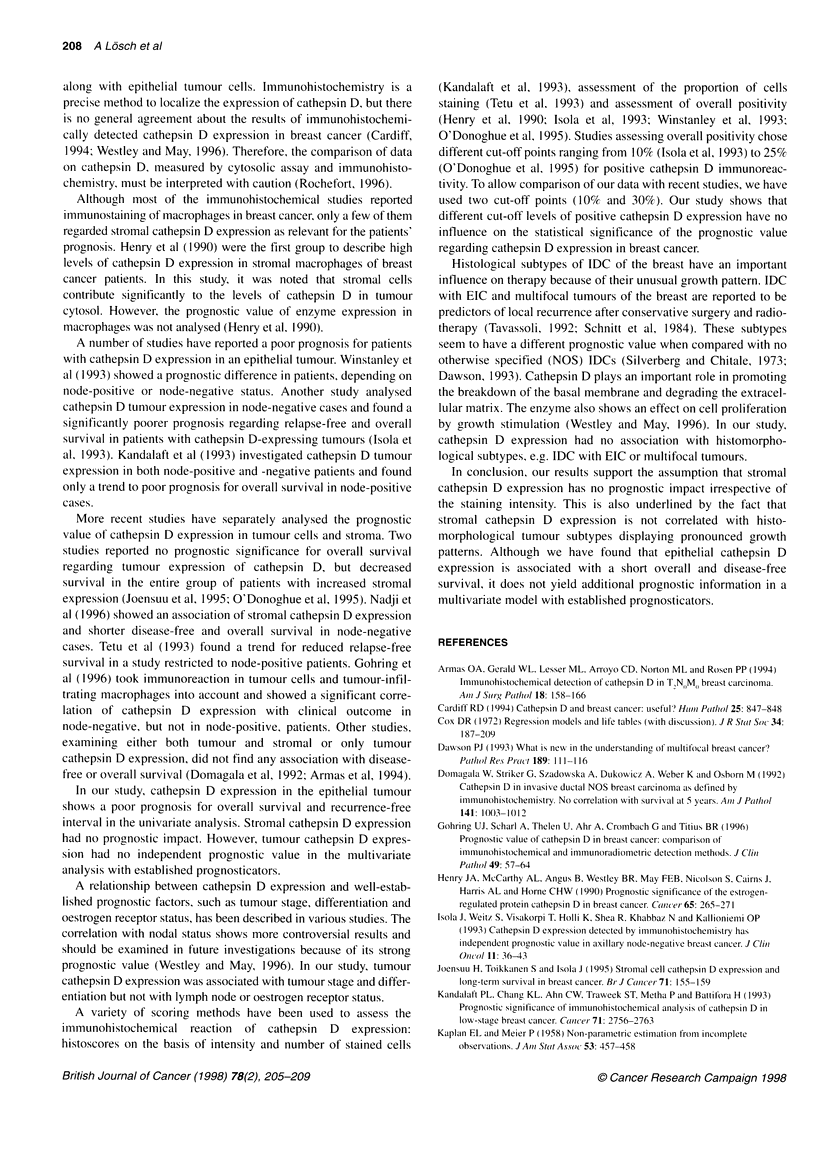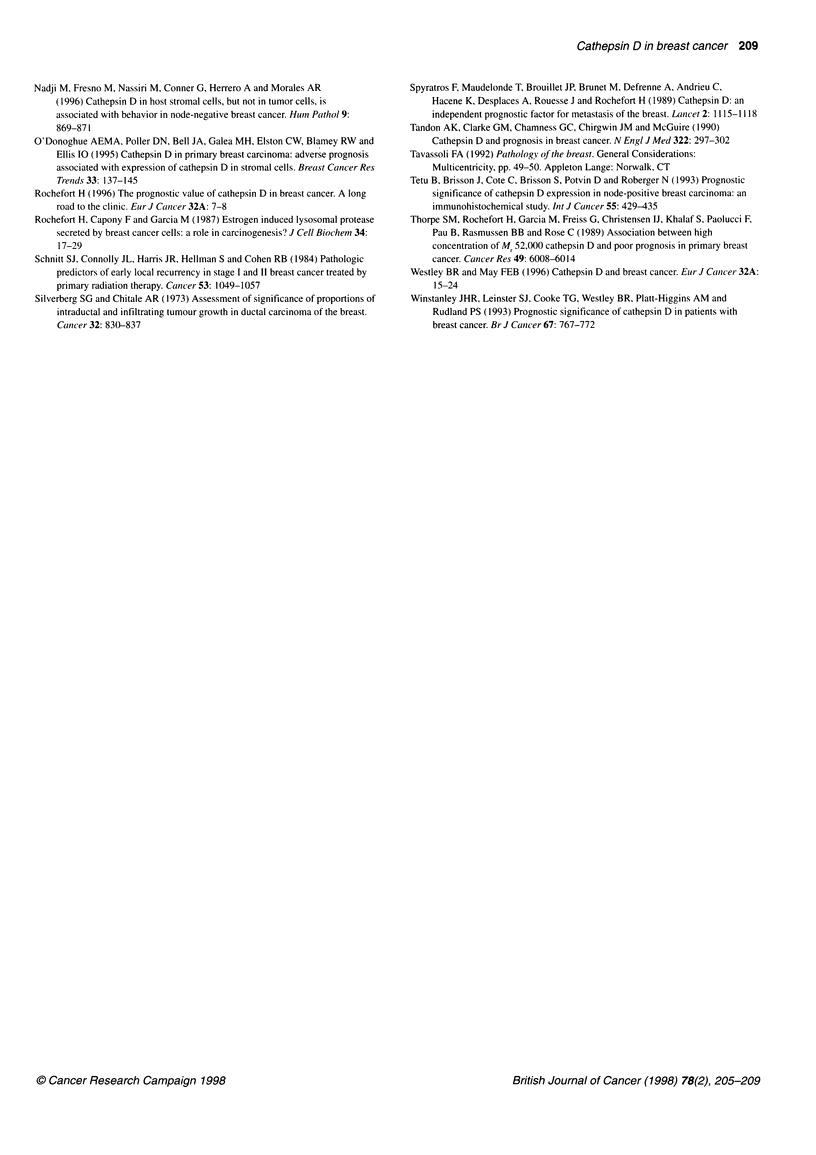# Prognostic value of cathepsin D expression and association with histomorphological subtypes in breast cancer.

**DOI:** 10.1038/bjc.1998.465

**Published:** 1998-07

**Authors:** A. Lösch, C. Tempfer, P. Kohlberger, E. A. Joura, M. Denk, B. Zajic, G. Breitenecker, C. Kainz

**Affiliations:** Gynaecopathological Unit, Institute of Pathology, University of Vienna, Austria.

## Abstract

This study investigated the prognostic value of immunohistochemically detected cathepsin D expression in 103 invasive ductal carcinomas of the breast at stages pT1 and 2. We also assessed the association between cathepsin D expression and histomorphological tumour subtypes (invasive ductal carcinoma with extensive intraductal component, multifocal tumour). Cathepsin D expression was examined at two cut-off levels (positive and highly positive) and separately identified within the epithelial and stromal component of all tumours. Positive and highly positive epithelial expression was detected in 32 (31.1%) and 20 (19.4%) patients respectively. Stromal expression was found in 35 (34%) and 19 (18.4%) cases respectively. Epithelial cathepsin D expression was associated with stage and nuclear grade, but not with lymph node or oestrogen receptor status. Positive and highly positive epithelial cathepsin D expression showed significant prognostic value for overall survival (P = 0.003 and 0.01) and recurrence-free interval (P = 0.04 and 0.02). Cathepsin D expression in stromal cells was not associated with either several established prognostic factors or survival. Multivariate analysis revealed that cathepsin D expression failed to be an independent predictor of patients' outcome. Cathepsin D expression shows no significant association with histomorphological subtypes of breast cancer. Our study supports the prognostic impact of immunohistochemically detected cathepsin D expression in the epithelial component of breast cancer.


British Journal of Cancer (1998) 78(2), 205–209
© 1998 Cancer Research Campaign

### Prognostic value of cathepsin D expression and association with histomorphological subtypes in breast cancer

**A Lösch[1], C Tempfer[2], P Kohlberger[2], EA Joura[2], M Denk[3], B Zajic[3], G Breitenecker[1] and C Kainz[2]**

[1]Gynaecopathological Unit, Institute of Pathology, [2]Department of Gynaecology and Obstetrics, Universitätsfrauenklinik, and [3]Department of Medical Computer Sciences, University of Vienna, Währinger Gürtel 18–20, A-1090 Vienna, Austria

**Summary** This study investigated the prognostic value of immunohistochemically detected cathepsin D expression in 103 invasive ductal carcinomas of the breast at stages pT1 and 2. We also assessed the association between cathepsin D expression and histomorphological tumour subtypes (invasive ductal carcinoma with extensive intraductal component, multifocal tumour). Cathepsin D expression was examined at two cut-off levels (positive and highly positive) and separately identified within the epithelial and stromal component of all tumours. Positive and highly positive epithelial expression was detected in 32 (31.1%) and 20 (19.4%) patients respectively. Stromal expression was found in 35 (34%) and 19 (18.4%) cases respectively. Epithelial cathepsin D expression was associated with stage and nuclear grade, but not with lymph node or oestrogen receptor status. Positive and highly positive epithelial cathepsin D expression showed significant prognostic value for overall survival ($P = 0.003$ and 0.01) and recurrence-free interval ($P = 0.04$ and 0.02). Cathepsin D expression in stromal cells was not associated with either several established prognostic factors or survival. Multivariate analysis revealed that cathepsin D expression failed to be an independent predictor of patients' outcome. Cathepsin D expression shows no significant association with histomorphological subtypes of breast cancer. Our study supports the prognostic impact of immunohistochemically detected cathepsin D expression in the epithelial component of breast cancer.

**Keywords:** cathepsin D; breast; neoplasm; prognosis

Cathepsin D is a ubiquitous lysosomal protease with important functions in protein catabolism. Three forms of the enzyme are known: 52 kDa procathepsin D, a 48-kDa intermediate form and stable cathepsin D with a 34-kDa heavy and a 14-kDa light chain. Oestrogen induces cathepsin D expression in breast cancer cells (Rochefort et al, 1987). The enzyme plays a key role in metastatic spread by promoting the destruction of normal tissue architecture and in tumour growth by the influence of growth factors (Westley and May, 1996). The prognostic value of cathepsin D in breast cancer is still controversial. Although most authors agree that high cathepsin D levels have a negative prognostic impact, the prognostic value of cathepsin D in clinically relevant subgroups of breast cancer patients has not been established (Rochefort, 1996). Furthermore, the importance of epithelial vs stromal expression of cathepsin D within the tumour is unclear. The prognostic value of cathepsin D expression in the various cell types within a tumour remains to be established in further investigations (Cardiff, 1994; Westley and May, 1996).

The cytosolic assay is a well-standardized method for the quantification of enzyme expression. The immunohistochemical method is not yet established for standardized quantification. However, this method allows a differentiation between tumour, stromal and non-tumour epithelial cell expression of cathepsin D.

*Received 7 October 1997*
*Revised 12 January 1998*
*Accepted 30 January 1998*

*Correspondence to*: A Lösch, Gynaecopathological Unit, Institute of Pathology, University of Vienna Medical School, AKH Vienna, Währinger Gürtel 18–20, AKH Vienna, A-1090 Vienna, Austria

The aim of this study was to investigate the prognostic value of immunohistochemically detected cathepsin D expression in tumour vs stromal tissue of 103 patients with invasive ductal carcinomas of the breast. Furthermore, we examined whether different cut-off points of immunoreactivity, which have been reported in previous studies, influence the prognostic significance of cathepsin D.

Several histomorphological subtypes of breast cancer display distinct growth patterns and behaviours of invasion (Tavassoli, 1992). In our study, we also examined the association between cathepsin D expression and the histomorphological subtypes infiltrating ductal cancer (IDC) with extensive intraductal component (EIC) and multifocal breast cancer.

### MATERIAL AND METHODS

We investigated 103 paraffin-embedded tumour specimens from women with primary invasive ductal carcinoma of the breast at stages pT1 and 2. During the period 1980–87, all patients with stage pT1 tumours underwent primary surgical treatment, including conservative tumour excision, and radiotherapy and all patients with stage pT2 tumours underwent radical mastectomy and radiotherapy. Axillary dissection was performed in all patients. In patients with positive lymph node status, adjuvant chemotherapy was administered after radical surgery. Anti-oestrogenic agents were given in patients with positive oestrogen receptor status. The mean age of patients was 54 years (range 23–81 years). Forty-two patients were premenopausal, whereas 61 were post-menopausal.

**Table 1** Correlation of positive cathepsin D expression (CD+) and high cathepsin D immunoreactivity (CD++) in the epithelial tumour (TU) or tumour-associated stroma (ST) of invasive ductal carcinomas of the breast with histological stage, differentiation, lymph node and oestrogen receptor status, presence of extensive intraductal tumour component (EIC) and multifocal appearance

| | *n* | CD+ TU | *P*-value | CD++ TU | *P*-value | CD+ ST | *P*-value | CD++ ST | *P*-value |
|---|---|---|---|---|---|---|---|---|---|
| Histological stage | | | | | | | | | |
| pT1 | 59 | 22.0% | 0.02 | 11.9% | 0.002 | 31.5% | 0.4 | 20.3% | 0.6 |
| pT2 | 44 | 43.2% | | 29.5% | | 38.6% | | 15.9% | |
| Histological grade | | | | | | | | | |
| Low (G1) | 27 | 14.8% | 0.03 | 3.7% | 0.02 | 25.9% | 0.3 | 22.2% | 0.6 |
| High (G2/3) | 76 | 36.8% | | 25.0% | | 40.8 | | 17.1% | |
| Nodal status | | | | | | | | | |
| Negative | 70 | 27.1% | 0.2 | 18.6% | 0.8 | 31.4% | 0.4 | 18.6% | 0.9 |
| Positive | 33 | 39.4% | | 21.2% | | 39.4% | | 18.2% | |
| Oestrogen receptor | | | | | | | | | |
| Negative | 63 | 37.0% | 0.7 | 22.2% | 0.9 | 29.6% | 0.5 | 14.8% | 0.2 |
| Positive | 40 | 32.4% | | 23.5% | | 44.1% | | 26.5% | |
| EIC | | | | | | | | | |
| Absent | 75 | 32.9% | 0.5 | 19.2% | 0.8 | 37.0% | 0.2 | 45.2% | 0.6 |
| Present | 28 | 25.9% | | 18.5% | | 22.2% | | 29.6% | |
| Tumour | | | | | | | | | |
| Unifocal | 85 | 32.9% | 0.4 | 21.2% | 0.6 | 36.5% | 0.2 | 20.0% | 0.7 |
| Multifocal | 18 | 22.2% | | 11.1% | | 22.2% | | 11.1% | |

Breast and axillary tissue sections were reviewed for tumour type, stage, grade, nodal status, presence of EIC and multifocal appearance of the tumour by two pathologists blinded to clinical data. According to the guidelines by Schnitt et al (1984), patients with a combination of intraductal carcinoma comprising 25% or more of the area encompassed by the IDC and intraductal carcinoma in the adjacent tissue were regarded as having tumours with EIC. Multifocal tumours were diagnosed according to the definition by Tavassoli (1992). Oestrogen receptor status was evaluated by immunoassay.

The median follow-up time was 78 months (range 11–172 months). During the observation period, 23 patients developed locoregional recurrence and eight showed distant metastases. Four patients had local recurrence and distant metastasis. Twenty-seven patients died from the disease.

### Immunohistochemistry

We performed immunohistochemistry using the primary antibody to cathepsin D (Dako Polyclonal rabbit anti-cathepsin D; code no. A561, Dako, Carpinteria, CA, USA). The specificity of this antibody was determined in a Western blot against purified cathepsin B, H, L and D. The antibody showed no reaction with cathepsin B, H or L. The antibody recognizes the 52-kDa precursor (procathepsin D) and the 48-kDa intermediate, active form of cathepsin D. The intracellular staining of the antibody proves immunoreactivity of the precursor and of the activated form of the enzyme in the cytoplasm.

All sections tested were routine formalin-fixed paraffin-embedded samples. Paraffin sections were soaked in xylene to remove paraffin and rehydrated in graded alcohol series (100% to 70%). To recover antigenicity, we used the 'Antigen Retrieval System' (BioGenex, San Ramon, CA, USA) twice for 5 min in a microwave (HM 146, Elektra Bregenz, Schwaz, Austria) on high power (650 W). The sections were then washed in 10 mM phosphate-buffered saline (PBS) (pH 7.6). The sections were incubated with cathepsin D antiserum at 1:300 dilution for 60 min at room temperature and then for a further 30 min with biotinylated anti-mouse and anti-rabbit link-antibody (Dako LSAB 2 Kit). After rinsing in PBS, the sections were coated with streptavidin conjugated to alkaline phosphatase for 10 min. The sections were rinsed in PBS, incubated with Fast Red chromogen (naphthol phosphate substrate in Tris buffer, 5 mg Fast Red chromogen tablets, BioGenex) and then washed in distilled water. The sections were finally counterstained with haematoxylin and mounted. The staining reaction was confined to the cytoplasm.

Control for the immunohistochemical reaction was performed once in every staining run. Localization of the immunohistochemical reaction and staining intensity was positive to the same degree in all positive controls. The positive control slide was prepared

**Table 2** Cathepsin D in invasive ductal carcinoma of the breast. Univariate analysis for overall survival, recurrence-free interval, pathological stage, histological grade and nodal status. Multivariate analysis for the prognostic value of overall survival using the generalized Cox models including pathological stage, histological grade and nodal status with the candidates: positive cathepsin D (CD+) and high cathepsin D immunoreactivity (CD++) in the epithelial tumour (TU) or the tumour-associated stroma (ST)

| | Univariate analysis | | Multivariate analysis | |
|---|---|---|---|---|
| | Recurrence-free interval (*P*-value) | Overall survival (*P*-value) | Overall survival RR[a] (95% CI[b]) | Overall survival (*P*-value) |
| Histological stage | 0.02 | 0.0001 | 3.9 (1.5–10.3) | 0.004 |
| Histological grade | 0.006 | 0.0002 | Monotone likelihood | 0.0001 |
| Nodal status | 0.007 | 0.0001 | 2.8 (1.3–6.3) | 0.01 |
| CD+/TU | 0.04 | 0.003 | 1.1 (0.4–3.0) | 0.9 |
| CD+/ST | 0.2 | 0.06 | 1.7 (0.7–4.4) | 0.3 |
| CD++/TU | 0.02 | 0.01 | 1.4 (0.6–3.3) | 0.4 |
| CD++/ST | 0.7 | 0.08 | 1.3 (0.5–3.5) | 0.6 |

[a]Relative risk. [b]Confidence interval.

© Cancer Research Campaign 1998

from breast skin tissue. In skin, the antibody labels sweat ducts and glands. In the subcutaneous tissue, the antibody stains normal myoepithelial cells of non-lactating mammary glands and cells of epithelial hyperplasia or apocrine metaplasia within areas of fibrocystic change. Macrophages, fibroblasts and lymphocytes showed occasional staining. The negative control slide was prepared from the same tissue block as the specimen. Instead of using a primary antibody, we used a non-immune rabbit serum (Dako code no. X902, 69 mg $ml^{-1}$) diluted 1:600.

We used a semiquantitative method to determine immunoreactivity. Epithelial tumour and stromal cell immunoreactivity were scored separately. The stromal component contained predominantly tumour-associated macrophages, fibroblasts and lymphoid cells. Immunoreactivity of $\leq$ 10% of the tumour or stromal cells was regarded as negative, immunoreactivity of > 10% was regarded as positive. Positive staining reaction in > 30% was referred to as high immunoreactivity. Each slide was examined by two pathologists. There was an interobserver variability in the assessment of staining. Agreement of the two observers occurred in 93% of the slides, which represents a high degree of consistency between the observers.

### Statistical analysis

Where appropriate, results were analysed by the chi-squared test. We calculated the survival and recurrence-free interval probabilities by the product limit method of Kaplan and Meier (Kaplan and Meier, 1958). Univariate analysis was assessed using the log-rank test. For multivariate analysis, the Cox proportional hazards model (Cox, 1972) was used to assess the independent effect of cathepsin D expression. The potential prognostic factor was added to a model of known prognostic factors: pathological stage, histological grade and lymph node status. The specific breast cancer-related survival was used in all analyses. Recurrence-free interval was defined as the time elapsed between the primary surgical treatment and the first verified metastasis or recurrence. All $P$-values are results of two-sided tests. The BMDP statistical software system (BMDP Statistical Software, Los Angeles, CA, USA, 1990) was used. $P$-values < 0.05 were considered statistically significant.

### RESULTS

Of the 103 IDCs of the breast studied, stage pT1 was present in 59 tumours (57.3%) and pT2 in 44 tumours (42.7%). We found low- (G1), moderate- (G2) and high-grade (G3) tumours in 26.2%, 36.9% and 36.9% respectively. To simplify the statistical analysis, grade 1 constituted the low-grade category, while nuclear grades 2 and 3 were combined into a high-grade category. Thirty-three patients (32%) had a positive histological lymph node status. IDC with EIC was diagnosed in 28 tumours (27.2%). Eighteen patients (17.5%) showed multifocal tumours. Seven patients (6.8%) presented with both histomorphological subtypes of breast cancer. Oestrogen receptor status was positive in 40 (38.8%) and negative in 63 (61.2%) cases.

We found positive granular, intracytoplasmic cathepsin D immunostaining reaction in 32 epithelial tumours (31.1%) and in 35 stromal components of the tumour (34%). High immunoreactivity was evident in 20 epithelial tumours (19.4%) and in 19 stromal components (18.4%). Sixty cases (58.3%) did not stain. Twenty-four cases (23.3%) showed expression of cathepsin D in both the tumour and the surrounding stroma; high immunoreactivity was found in six of these cases (5.8%).

**Figure 1** Kaplan–Meier overall survival analysis of patients with positive (CD+, log-rank $P$ = 0.003) and high cathepsin D (CD++, log-rank $P$ = 0.01) epithelial tumour expression compared with patients with negative results (CD —, CD – –)

The statistical results of carcinomas with positive cathepsin D expression were compared with cases with high immunoreactivity. There was no difference in the statistical significance between both groups regarding the association of cathepsin D expression with established prognostic factors, IDC with EIC and multifocal tumours (Table 1), and prognosis for overall survival and recurrence-free interval (Table 2).

The results of cathepsin D expression with tumour stage, grade, nodal status, oestrogen receptor status, IDC with EIC and multifocal tumours are presented in Table 1. Data from the univariate and multivariate analysis regarding overall survival and recurrence-free interval are shown in Table 2. The number of patients was too small to consider pre- and postmenopausal or lymph node-positive or -negative patients in a subgroup analysis. The survival distribution function grouped by cathepsin D epithelial tumour expression is shown in Figure 1.

### DISCUSSION

Most biochemical studies of cathepsin D in breast cancer show that elevated total tumour cathepsin D is an adverse prognostic factor. Subgroup analysis regarding node-negative and node-positive tumours yielded controversial results (Spyratros et al, 1989; Thorpe et al, 1989; Tandon et al, 1990; Westley and May, 1996). The cytosolic assay is an established method for quantification of total enzyme expression without differentiation between benign or malignant, epithelial or stromal cell types. During recent years the importance of the tumour cell stroma in the process of breast cancer invasion has become more evident. Several proteases have been shown to be active in stromal cells of malignant tumours (Joensuu et al, 1995). In breast cancer, stromal cells such as tumour-associated macrophages can overexpress cathepsin D

© Cancer Research Campaign 1998

along with epithelial tumour cells. Immunohistochemistry is a precise method to localize the expression of cathepsin D, but there is no general agreement about the results of immunohistochemically detected cathepsin D expression in breast cancer (Cardiff, 1994; Westley and May, 1996). Therefore, the comparison of data on cathepsin D, measured by cytosolic assay and immunohistochemistry, must be interpreted with caution (Rochefort, 1996).

Although most of the immunohistochemical studies reported immunostaining of macrophages in breast cancer, only a few of them regarded stromal cathepsin D expression as relevant for the patients' prognosis. Henry et al (1990) were the first group to describe high levels of cathepsin D expression in stromal macrophages of breast cancer patients. In this study, it was noted that stromal cells contribute significantly to the levels of cathepsin D in tumour cytosol. However, the prognostic value of enzyme expression in macrophages was not analysed (Henry et al, 1990).

A number of studies have reported a poor prognosis for patients with cathepsin D expression in an epithelial tumour. Winstanley et al (1993) showed a prognostic difference in patients, depending on node-positive or node-negative status. Another study analysed cathepsin D tumour expression in node-negative cases and found a significantly poorer prognosis regarding relapse-free and overall survival in patients with cathepsin D-expressing tumours (Isola et al, 1993). Kandalaft et al (1993) investigated cathepsin D tumour expression in both node-positive and -negative patients and found only a trend to poor prognosis for overall survival in node-positive cases.

More recent studies have separately analysed the prognostic value of cathepsin D expression in tumour cells and stroma. Two studies reported no prognostic significance for overall survival regarding tumour expression of cathepsin D, but decreased survival in the entire group of patients with increased stromal expression (Joensuu et al, 1995; O'Donoghue et al, 1995). Nadji et al (1996) showed an association of stromal cathepsin D expression and shorter disease-free and overall survival in node-negative cases. Tetu et al (1993) found a trend for reduced relapse-free survival in a study restricted to node-positive patients. Gohring et al (1996) took immunoreaction in tumour cells and tumour-infiltrating macrophages into account and showed a significant correlation of cathepsin D expression with clinical outcome in node-negative, but not in node-positive, patients. Other studies, examining either both tumour and stromal or only tumour cathepsin D expression, did not find any association with disease-free or overall survival (Domagala et al, 1992; Armas et al, 1994).

In our study, cathepsin D expression in the epithelial tumour shows a poor prognosis for overall survival and recurrence-free interval in the univariate analysis. Stromal cathepsin D expression had no prognostic impact. However, tumour cathepsin D expression had no independent prognostic value in the multivariate analysis with established prognosticators.

A relationship between cathepsin D expression and well-established prognostic factors, such as tumour stage, differentiation and oestrogen receptor status, has been described in various studies. The correlation with nodal status shows more controversial results and should be examined in future investigations because of its strong prognostic value (Westley and May, 1996). In our study, tumour cathepsin D expression was associated with tumour stage and differentiation but not with lymph node or oestrogen receptor status.

A variety of scoring methods have been used to assess the immunohistochemical reaction of cathepsin D expression: histoscores on the basis of intensity and number of stained cells (Kandalaft et al, 1993), assessment of the proportion of cells staining (Tetu et al, 1993) and assessment of overall positivity (Henry et al, 1990; Isola et al, 1993; Winstanley et al, 1993; O'Donoghue et al, 1995). Studies assessing overall positivity chose different cut-off points ranging from 10% (Isola et al, 1993) to 25% (O'Donoghue et al, 1995) for positive cathepsin D immunoreactivity. To allow comparison of our data with recent studies, we have used two cut-off points (10% and 30%). Our study shows that different cut-off levels of positive cathepsin D expression have no influence on the statistical significance of the prognostic value regarding cathepsin D expression in breast cancer.

Histological subtypes of IDC of the breast have an important influence on therapy because of their unusual growth pattern. IDC with EIC and multifocal tumours of the breast are reported to be predictors of local recurrence after conservative surgery and radiotherapy (Tavassoli, 1992; Schnitt et al, 1984). These subtypes seem to have a different prognostic value when compared with no otherwise specified (NOS) IDCs (Silverberg and Chitale, 1973; Dawson, 1993). Cathepsin D plays an important role in promoting the breakdown of the basal membrane and degrading the extracellular matrix. The enzyme also shows an effect on cell proliferation by growth stimulation (Westley and May, 1996). In our study, cathepsin D expression had no association with histomorphological subtypes, e.g. IDC with EIC or multifocal tumours.

In conclusion, our results support the assumption that stromal cathepsin D expression has no prognostic impact irrespective of the staining intensity. This is also underlined by the fact that stromal cathepsin D expression is not correlated with histomorphological tumour subtypes displaying pronounced growth patterns. Although we have found that epithelial cathepsin D expression is associated with a short overall and disease-free survival, it does not yield additional prognostic information in a multivariate model with established prognosticators.

### REFERENCES

Armas OA, Gerald WL, Lesser ML, Arroyo CD, Norton ML and Rosen PP (1994) Immunohistochemical detection of cathepsin D in $T_2N_0M_0$ breast carcinoma. *Am J Surg Pathol* **18**: 158–166

Cardiff RD (1994) Cathepsin D and breast cancer: useful? *Hum Pathol* **25**: 847–848

Cox DR (1972) Regression models and life tables (with discussion). *J R Stat Soc* **34**: 187–209

Dawson PJ (1993) What is new in the understanding of multifocal breast cancer? *Pathol Res Pract* **189**: 111–116

Domagala W, Striker G, Szadowska A, Dukowicz A, Weber K and Osborn M (1992) Cathepsin D in invasive ductal NOS breast carcinoma as defined by immunohistochemistry. No correlation with survival at 5 years. *Am J Pathol* **141**: 1003–1012

Gohring UJ, Scharl A, Thelen U, Ahr A, Crombach G and Titius BR (1996) Prognostic value of cathepsin D in breast cancer: comparison of immunohistochemical and immunoradiometric detection methods. *J Clin Pathol* **49**: 57–64

Henry JA, McCarthy AL, Angus B, Westley BR, May FEB, Nicolson S, Cairns J, Harris AL and Horne CHW (1990) Prognostic significance of the estrogen-regulated protein cathepsin D in breast cancer. *Cancer* **65**: 265–271

Isola J, Weitz S, Visakorpi T, Holli K, Shea R, Khabbaz N and Kallioniemi OP (1993) Cathepsin D expression detected by immunohistochemistry has independent prognostic value in axillary node-negative breast cancer. *J Clin Oncol* **11**: 36–43

Joensuu H, Toikkanen S and Isola J (1995) Stromal cell cathepsin D expression and long-term survival in breast cancer. *Br J Cancer* **71**: 155–159

Kandalaft PL, Chang KL, Ahn CW, Traweek ST, Metha P and Battifora H (1993) Prognostic significance of immunohistochemical analysis of cathepsin D in low-stage breast cancer. *Cancer* **71**: 2756–2763

Kaplan EL and Meier P (1958) Non-parametric estimation from incomplete observations. *J Am Stat Assoc* **53**: 457–458

© Cancer Research Campaign 1998

Nadji M, Fresno M, Nassiri M, Conner G, Herrero A and Morales AR (1996) Cathepsin D in host stromal cells, but not in tumor cells, is associated with behavior in node-negative breast cancer. *Hum Pathol* **9**: 869–871

O'Donoghue AEMA, Poller DN, Bell JA, Galea MH, Elston CW, Blamey RW and Ellis IO (1995) Cathepsin D in primary breast carcinoma: adverse prognosis associated with expression of cathepsin D in stromal cells. *Breast Cancer Res Trends* **33**: 137–145

Rochefort H (1996) The prognostic value of cathepsin D in breast cancer. A long road to the clinic. *Eur J Cancer* **32A**: 7–8

Rochefort H, Capony F and Garcia M (1987) Estrogen induced lysosomal protease secreted by breast cancer cells: a role in carcinogenesis? *J Cell Biochem* **34**: 17–29

Schnitt SJ, Connolly JL, Harris JR, Hellman S and Cohen RB (1984) Pathologic predictors of early local recurrency in stage I and II breast cancer treated by primary radiation therapy. *Cancer* **53**: 1049–1057

Silverberg SG and Chitale AR (1973) Assessment of significance of proportions of intraductal and infiltrating tumour growth in ductal carcinoma of the breast. *Cancer* **32**: 830–837

Spyratros F, Maudelonde T, Brouillet JP, Brunet M, Defrenne A, Andrieu C, Hacene K, Desplaces A, Rouesse J and Rochefort H (1989) Cathepsin D: an independent prognostic factor for metastasis of the breast. *Lancet* **2**: 1115–1118

Tandon AK, Clarke GM, Chamness GC, Chirgwin JM and McGuire (1990) Cathepsin D and prognosis in breast cancer. *N Engl J Med* **322**: 297–302

Tavassoli FA (1992) *Pathology of the breast*. General Considerations: Multicentricity, pp. 49–50. Appleton Lange: Norwalk, CT

Tetu B, Brisson J, Cote C, Brisson S, Potvin D and Roberger N (1993) Prognostic significance of cathepsin D expression in node-positive breast carcinoma: an immunohistochemical study. *Int J Cancer* **55**: 429–435

Thorpe SM, Rochefort H, Garcia M, Freiss G, Christensen IJ, Khalaf S, Paolucci F, Pau B, Rasmussen BB and Rose C (1989) Association between high concentration of $M_r$ 52,000 cathepsin D and poor prognosis in primary breast cancer. *Cancer Res* **49**: 6008–6014

Westley BR and May FEB (1996) Cathepsin D and breast cancer. *Eur J Cancer* **32A**: 15–24

Winstanley JHR, Leinster SJ, Cooke TG, Westley BR, Platt-Higgins AM and Rudland PS (1993) Prognostic significance of cathepsin D in patients with breast cancer. *Br J Cancer* **67**: 767–772

© Cancer Research Campaign 1998